# Rochelle salt – a structural reinvestigation with improved tools. I. The high-temperature para­electric phase at 308 K

**DOI:** 10.1107/S2052252514022155

**Published:** 2015-01-01

**Authors:** Frode Mo, Ragnvald H. Mathiesen, Jon Are Beukes, Khanh Minh Vu

**Affiliations:** a Department of Physics, Norwegian University of Science and Technology, Høgskoleringen 5, N-7491 Trondheim, Norway

**Keywords:** single-crystal X-ray study, synchrotron radiation, control of sample environment

## Abstract

The crystal structure of the high-temperature paraelectric phase of Rochelle salt (K^+^·Na^+^·C_4_H_4_O_6_^2−^·4H_2_O) at 308 K has been reinvestigated using synchrotron X-ray diffraction with refinement parameters *R*(int) = 0.0123, final (shift/e.s.d.)_max_ = 0.019, *R*_1_(all) = 0.0371 and *wR*_2_(all) = 0.0608. The application of a new gas-flow sample cell designed to control both temperature and relative humidity permitted collection of data of excellent quality and enabled unrestrained refinement of all parameters, including those of the isotropic hydrogen atoms. A precise description of the structure has ensued. One K atom is disordered between two symmetry-equivalent sites; three O atoms in three of the four water molecules exhibit very strong anisotropy. Refining one O atom as a split atom was successful, yielding small improvements in the bonding parameters of several H atoms. The H atoms of all water molecules behave as single pairs. Their final *U* values are of moderate magnitude indicating that these atoms do not participate in the anisotropy of the parent O atoms. It is suggested that the three water O atoms are in part statically disordered, while the bonded H atoms are not. Except for the split K atom and the three water O atoms there is no evidence of general disorder in the structure.

## Abstract 2

A novel sample cell with control of temperature and relative humidity permitted collection of data of excellent quality, enabling unrestrained refinement of all atomic parameters. One of the K atoms in the structure is disordered; very strong anisotropy in three of the four water O atoms indicates partial static disorder which does not involve the bonded H atoms.

## 1. Introduction   

Rochelle salt (KNaC_4_H_4_O_6_·4H_2_O) was apparently first manufactured in 1665 by Elie Seignette, an apothecary in La Rochelle (France), in an effort to make a purgative drug with minimal side effects (Lüker, 2009 ▶). Rochelle salt, also called Seignette salt, was very successfully used for more than 200 years as a mild purgative, and as well for various chemical purposes. The extraordinary physical properties of this salt were beginning to emerge from studies in the nineteenth century. In Scotland, the phenomenon of pyroelectricity in some inorganic crystals, among them Rochelle salt (RS), was observed by Brewster (1824 ▶). The brothers Jacques and Pierre Curie in famous works (Curie & Curie, 1880*a* ▶,*b* ▶,*c* ▶) established unequivocally the existence of a piezoelectric effect, and correctly identified RS and several other crystals as being piezoelectric. Presumably one of the very first studies of the anomalously high dielectric response of RS was carried out by Pockels for his dissertation work (Pockels, 1894 ▶). Pockels also discovered a very large irreversible electro-optical Kerr effect for electric fields parallel to the *a* axis of this salt (Pockels, 1906 ▶). From analogies between the then established theory for paramagnetism and the novel phenomenon of piezoelectricity, Debye (1912 ▶) hypothesized that the latter property must be due to a permanent electric dipole moment set up by the molecules in the crystal, and further that there must be a critical temperature for which the dielectric constant goes to infinity, hence an analogy with the Curie temperature of a ferromagnet. Major contributions to the characterization of RS were made by Valasek (1921 ▶, 1922*a* ▶,*b* ▶), who also drew on analogies, in this case between the electrical properties of RS and the magnetic properties of iron. He provided the first experimental evidence of a hysteresis in polarization as a function of the sign and the magnitude of an electric field, and mapped out the piezoelectric response to temperature, revealing the existence of high piezoelectric activity in a narrow range in *T*, from about 253 to 298 K, with a maximum at about 273 K; thus indicating the occurrence of two phase transitions and two Curie points which are indeed a peculiar property of RS crystals. The term ferroelectricity that came into use later was for many years singularly linked to RS until about 1935 when the first inorganic ferroelectric materials were discovered. A summing-up of the experimental evidence that had been acquired for RS by then was given by Mueller in a series of papers (Mueller, 1935 ▶, 1940*a* ▶,*b* ▶,*c* ▶). Mueller also discussed four possible theories for its anomalous dielectric properties and the relative merits of these theories (Mueller, 1940*a* ▶). At the time, the crystal structure of RS was not known. This information is required for the analysis of its phase transformations.

The exceptionally large piezoelectric response of RS has been put to use, in particular, in electromechanical transducers *e.g.* in various appliances for the detection and generation of sonic and supersonic waves. Several examples can be found in the books by Cady (1946 ▶) and Mason (1950 ▶). A serious difficulty affecting the application of RS is that the crystals are unstable and deteriorate easily, either by dehydration or by liquefaction, when exposed to relative humidity outside of the stable range.

RS in its ferroelectric (FE) phase is monoclinic (*P*2_1_11, *Z* = 4) and is thermally bracketed by two Curie points, *T*_C1_ = 255 K and *T*_C2_ = 296 K. At both of these temperatures, a phase transition to a paraelectric structure of identical orthorhombic symmetry takes place (*P*2_1_2_1_2, *Z* = 4). The spontaneous polarization in the FE phase is directed along the *a* axis (Jona & Shirane, 1962 ▶).

The first single-crystal X-ray diffraction study of RS by Beevers & Hughes (1941 ▶) provided a preliminary structure (excluding H atoms) from projections along the main crystallographic axes. The phase transition was proposed to be associated with the polarizability of a hydrogen-bond linking carboxyl-oxygen O1 and water-oxygen O10 (in the atomic labelling scheme of Beevers & Hughes). This pioneering work has been succeeded by a large number of diffractions studies by other investigators.

Ubbelohde & Woodward (1946 ▶) elaborated further on the theory of Beevers & Hughes, in particular proposing that the onset of anomalous dielectric properties at the lower Curie point can be ascribed to thermal expansion of this hydrogen bond. From their neutron diffraction work, Frazer *et al.* (1954 ▶) obtained more reliable data for the hydrogen-bond network and found the existing explanation improbable. This and other diffraction studies revealing significant discrepancies from the Beevers–Hughes model indicating other hydrogen bonds being responsible for the phase transition were announced by Shirane *et al.* (1955 ▶), but the results apparently were never published. The three phases of RS have been re-examined in X-ray and neutron diffraction analyses by several authors (Mazzi *et al.*, 1957 ▶; Okaya *et al.*, 1960 ▶; Frazer, 1962 ▶; Shiozaki & Mitsui, 1972 ▶; Mitani *et al.*, 1974 ▶). The main results allegedly relating to the phase transition comprise, as the key feature, a local disorder model in which the H atom bonded to the hydroxyl-O atom O5 undergoes a reorientational motion, and in addition disorder in the water molecule H_2_O(8) or *W*8. Mazzi *et al.* (1957 ▶) found large anisotropy for some of the atoms, in particular for K1, and for the oxygen atoms O3, O8 and O9, the latter belonging to water molecule *W*9.

In subsequent neutron diffraction studies of the paraelectric (PE) structures at 78 and 313 K in which all atoms were refined with anisotropic displacement parameters (ADPs), Iwata (Iwata *et al.*, 1989 ▶; Iwata, Mitani & Shibuya, 1989 ▶, 1990 ▶) found no evidence of local disorder previously assigned to the O5—H bond. However, according to these authors nearly all atoms in the low-*T* and even more strongly so in the high-*T* structure exhibit distinct anisotropy, thus confirming and extending the observations of Mazzi *et al.* (1957 ▶). A split-atom model that included the tartrate molecules was tentatively used in the refinements in order to describe transition between two stable sites in the PE phases. A flip-flop motion along the polar *a* axis, or [100], was proposed for the water molecule *W*8 in the high-*T* PE structure. Results from X-ray studies of RS at 243, 273 and 308 K by Suzuki *et al.* (1994 ▶) and Suzuki & Shiozaki (1996 ▶) were interpreted as cooperative atomic displacements occurring at the onset of the spontaneous polarization. The displacements involve the tartrate molecules and the water molecules within a frame composed of the K and Na atoms. This theory has been further elaborated upon by Shiozaki and collaborators (Shiozaki *et al.*, 1998 ▶, 1999 ▶, 2001 ▶). They found that the PE structure, including K1, is disordered both at low and high *T* and describe the structure as a superposition of two equivalent sublattices of opposed polarity. These sublattices become nonequivalent in the FE phase giving rise to a spontaneous polarization. In contrast, Solans *et al.* (1997 ▶) from their X-ray studies of RS at 213, 274 and 323 K did not find evidence for disorder in the PE structures. These authors proposed a model in which the high- and low-*T* structures, each comprising two chains related by the twofold axis parallel to [001] and each chain with a polarization vector **P**, give a net zero polarization along [100]. The polarization vector **P** is of different magnitude in the high-*T* and the low-*T* phases, **P**_HT_ and **P**_LT_, respectively. The FE phase can be viewed as an ordered solid solution of chains with polarization **P**_HT_ and others with **P**_LT_.

The question of whether the phase transitions are of the order–disorder or of the displacive type has remained elusive. The results from spectroscopy studies are ambiguous. Measurements of the complex dielectric constant of RS showed a distinct soft relaxation and a critical slowing down near the transition points indicating an order–disorder-type transition (Sandy & Jones, 1968 ▶). On the other hand, the soft mode observed in infrared reflectivity spectra by Raman scattering in the low-*T* PE phase (Kamba *et al.*, 1995 ▶) and in microwave dielectric measurements (Volkov *et al.*, 1986 ▶) indicate as more likely a displacive-type transition at *T_C_*_1_.

Great efforts have been made to give a theoretical description at the atomic level of the phase transitions in RS. An important model, from Mitsui (1958 ▶), is based on two interpenetrating sublattices and takes into account two key features: an asymmetry in population in pairs of local atomic positions, and compensation of the electric dipole moments in the PE phases. This model, which assumes an order–disorder-type transition, has been further extended and refined by many investigators [see Levitskii *et al.* (2009 ▶) and Moina *et al.* (2011 ▶)].

In conclusion, it can be stated that neither the experimental evidence acquired so far nor the theoretical models suffice to give a definite answer to the question of the nanoscopic nature of the phase transitions of RS. The transitions are linked to subtle structural changes involving many atoms to which belong at least three of the four water molecules. Interpretation of the observed large anisotropy in diffraction studies is difficult but crucial in the case of RS. It is clear that diffraction data of very high quality are required. However, the acquisition of such data is not trivial. The instability of RS crystals, in particular their facile dehydration, is one impediment. A second serious problem is that these crystals are easily damaged by X-ray radiation (Frazer, 1962 ▶; Boutin *et al.*, 1963 ▶; Shiozaki, 1967 ▶; Okada *et al.*, 1967 ▶). Both of these factors must be taken into account and minimized in the experimental work.

We describe here a crystallographic study of the high-*T* PE phase at 308 K using synchrotron radiation.

## 2. Experimental   

### 2.1. Sample preparation   

Crystallization of RS was carried out in a scaled-down version of a recipe given by Holden & Morrison (1982 ▶). Under heating on a water bath at 333 K, 19.5 g Rochelle salt (NaKC_4_H_4_O_6_·4H_2_O, *p.a.* quality from Merck) was dissolved in 15 ml distilled water. The supersaturated solution was covered and set aside for cooling to room temperature, then seeded with a few tiny crystal grains. The beaker was covered with a polymer film and left for crystallization. Large and nearly perfect crystals quickly started to grow from the solution and after about 1–1.5 h the crystals were harvested by filtering through a sintered glass filter. Samples of suitable size for diffraction work with synchrotron radiation were obtained by cutting them from larger specimens. Cutting of these hard crystals frequently introduced defects, as shown by broadened and split reflections. About one out of every three cut samples was of good quality for data collection.

### 2.2. Stability of the crystals   

RS effloresces on exposure to a warm and dry environment: at 328 K, it separates into sodium and potassium salts with the evolution of one molecule of water; at 373 K, in total three molecules of water will be lost. The mobile water molecules constitute a labile structural element that is activated under the influence of humidity and temperature, thereby inducing a structural reorganization. The loss of water is accompanied by the growth of a white crust of dehydrated material on the crystal surface. The partial changes in structure associated with the dehydration are detrimental for the acquisition of accurate diffraction data, which is needed in order to elucidate the allegedly subtle structural changes associated with the phase transitions involving water molecules and hydrogen bonds. In some previous studies of the PE phases, a single-crystal was placed in a sealed glass capillary (X-rays) (Suzuki & Shiozaki, 1996 ▶; Shiozaki *et al.*, 1998 ▶) or in an airtight aluminium container (neutrons) (Iwata *et al.*, 1989 ▶). However, in most studies the instability of RS crystals seems to have been ignored. In the X-ray case, we observe that the use of a glass capillary creates other problems, augmented if the crystal is large, due to large and strongly anisotropic absorption and, as well, end effects due to the smaller cross section of the beam. The combined effect may well impair significantly the collection of high-quality diffraction data. Moreover, if the relative humidity (RH) of the environment in the capillary is not within the safe range between efflorescence and deliquescence at the temperature *T* of the experiment, a gradual deterioration of the crystal will take place. A safe range in RH *versus T* can be found on pp. 115–117 in Mason (1950 ▶).

We have observed that the slow dehydration process taking place naturally in a dry environment is strongly promoted, and actually seems to accelerate under exposure to X-rays. We found that an unprotected crystal kept in a dry N_2_ atmosphere at 308 K and exposed to X-rays for about 1 h had transformed into a poorly scattering, white opaque lump after another 20 h without radiation. Coating the crystal with an inert oil improved its lifetime considerably, to about three days, however, with a gradual and increasing degradation during the last 15–20 h as judged from a set of measured standard reflections. The best results were obtained with an uncoated crystal kept in a stream of moist N_2_ gas under control of RH and temperature.

### 2.3. Sample environment, data collection and processing   

A new sample cell was designed to enable control of RH over a wide range (0–95%) and *T* in the range 263–323 K. The base of the cell is a ring-shaped collar screwed directly onto the locking ring of the goniometer head on the diffractometer. Heating or cooling is provided by Peltier elements arranged in an annular ring within the collar, which serves as the thermal exchange unit (TEU). Two streams of N_2_ gas, one dry and one humidified, are mixed in a fixed ratio to maintain an RH of the sample environment within the desired range. The gas mixture is cooled or heated as it diffuses through the TEU, exits and flows upward along the solid crystal mount that is surrounded by two conical Kapton shields. The gas leaves the outer Kapton cone through its open tip. Two small thermocouples, one placed on the exit side of the TEU, and the other mounted just below the crystal can be read continuously during the experiment. The secondary side of the Peltier elements can be cooled or heated as required with a fluid, in this case water that is led through a heat-exchanging circuit. Details of the design and performance of the cell with some additional features are given elsewhere (Mo & Ramsøskar, 2009 ▶). A capacitor for applying an electric field on the sample and depicted in this reference was not implemented for the present study of the high-*T* PE phase.

Synchrotron radiation with λ = 0.60097 Å was used for the experiment; intensities were measured using ω-scans with a scintillation detector on a KM-6 Kappa diffractometer (Thorkildsen *et al.*, 1999 ▶). All measurements were made at *T* = 308.0 (7) K with RH in the range 60–70%. Crystal orientation and unit-cell dimensions were determined before and towards the end of the data collection by measurements of up to 47 reflections, of which nearly 80% had sinθ/λ (= *s*) in the range 0.54–0.894 Å^−1^ (2θ range 38–65°). Unit-cell axis *b* had increased significantly (by ∼ 10σ) from 14.3037 (6) to 14.3094 (5) Å, during a period of irradiation of about 70 h. A smaller increase of 4σ was found in *c*, while *a* remained unchanged.

Because of physical constraints imposed by the sample cell and the associated leads for water and N_2_ gas, data collection in the hemisphere with *l* > 0 was difficult, requiring constant surveillance and frequent manual interference. Therefore, 98% of the data has *l* < 0. All reflections in the *s* range 0.0436–0.500 Å^−1^ (2θ: 3.0–35°) within this hemisphere were collected. Two quadrants were remeasured using a 1000 µm-thick Al attenuator foil, to accomplish interscaling and determination of an attenuation factor for the strong reflections. Two quadrants were collected in the range 0.492 < *s* < 0.781 Å^−1^ (2θ: 34.4–56°). Reflections in the 2θ range 3.0–56° were measured from two to ten times, as measurements of identical reflections, symmetry equivalents or Friedel pairs. There was a significant weakening of the intensities with increasing angle indicating large atomic displacements. At the upper limit in *s* = 0.781 Å^−1^ (*s*_high_), a large fraction of the reflections was weak. In order to save time, only reflections with intensities calculated above a selected limit (in total 586) were collected in the range 0.768 < *s* < 1.00 Å^−1^ (55 < 2θ < 74°). Three standard reflections were measured every 150 data reflections for control and subsequent scaling. During the data collection, reflections were repeatedly scaled and merged and the development of the standards was followed as an on-line quality control. This procedure was very useful, revealing temporary systematic discrepancies, *e.g.* following a beam dump that lasted for an hour or sometimes after a beam refill. ‘Bad’ patches of reflections were remeasured to replace the flawed measurements.

Throughout the measurement period that lasted about 77 h, full width at half-maximum values for three test reflections were checked at regular intervals using ω-scans. The initial values in the range 0.0055–0.007° did not increase, indicating no increase in mosaicity which would take place during dehydration. Except during temporary beam instabilities such as mentioned above, there were insignificant changes in the intensities of the three standard reflections until about two-thirds of the data had been collected, when a slow and uniform decrease started giving a final 10% reduction in intensity.

In total, 8963 data reflections were measured of which 210 collected during periods of beam instability were discarded and replaced by new measurements. All measurements were corrected for systematic variations in the incident beam based on the monitor readings, subsequently scaled according to a polynomial fit [*y*(*x*)] to the average standard curve, and corrected for Lorentz and polarization effects. Errors in the data were calculated from σ(*I*) = 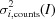 + (*S* *I*_net_)^2^, where *i* is the number of measurements for a given reflection. The parameter *S* was obtained from merging the data, adjusting *S* to obtain a normal distribution of the weighted means Δ*_i_* = |*F*_*i*_^2^ − *F*_w_^2^|. At the radiation energy of the experiment, 20.630 keV, the ratio between the imaginary and the real parts of the complex scattering factor, *f*_2_/*f*_1_, is 0.0099, or ∼1.0% for K, and significantly smaller for the other elements in the sample. The initial merging included Friedel pairs to obtain a sufficiently large number of data points for the determination of *S*. The number of reflections after merging was 2731, of which 258 had *F*_w_^2^ < σ(*F*_w_^2^) and were given weight *w* = 0. With *S* = 0.007 the merging indices, *R*_merge_(all) = 0.0158, *R*_merge_(obs) = 0.0153 and *R*_sigma_(all) = 0.0201.

A correction for thermal diffuse scattering (TDS) was applied to the data in the form *I*_corr_ = *I*/(1 + α), where α contains the correction due to one- and two-photon scattering. Coefficients for compliance and piezoelectric coupling were taken from the literature (http://www.efunda.com/materials/piezo/material_data/matdata_output.cfm?Material_ID=­Rochelle_Salt) and used to calculate the elastic constants *c_ij_*. Merging of the corrected data gave a small reduction in the indices, *R*_merge_(all) = 0.0157, *R*_merge_(obs) = 0.0152. The expected improvement of ADPs justifies the TDS correction of the data.

Finally the data were corrected for absorption (de Meulenaer & Tompa, 1965 ▶) from the shape of the crystal measured under a microscope. The final proper merging excluding Friedel mates gave *R*_merge_(all) = 0.0123, *R*_merge_(obs) = 0.0121 and *R*_sigma_(all) = 0.0146.

### 2.4. Structure refinement   

Non-hydrogen atom coordinates from the refinement at 313 K of the high-*T* form by Iwata, Mitani & Shibuya (1989 ▶) together with a uniform *U* = 0.04 Å^2^ were used as starting parameters. After the initial isotropic refinement K1 was found in a steep gradient with extrema +3.2/−2.0 e Å^−3^. Pairs of other residual maxima indicated strong anisotropy for the water oxygen atoms O10 and O8 and after subsequent refinement cycles for water oxygen O9 also. The features near K1 could not be described with ADPs so a disorder model had to be employed for this atom, thereby shifting it from the special position 0, 0, *z*. All H atoms were located successively from Δ*F* maps in peaks of density in the range 0.3–0.65 e Å^−3^. Refinement of all anisotropic non-H atoms and isotropic H atoms was carried out without restraints and converged at *wR*(*F*_o_^2^) = 0.0716 for 4711 *F*_o_^2^, *R*(*F*_o_) = 0.0266 for 3879 *F*_o_ > 4σ(*F*_o_) and *R*(*F*_o_) = 0.0402 for all 4711 *F*_o_. The largest residual features at this stage occurred near O10 which was located in an elongated maximum of density 0.39 e Å^−3^, with a pair of extended minima of density −0.39 e Å^−3^ on either side. A split model with isotropic displacement parameters was introduced in order to improve the description of this atom. Following anisotropic refinement of the pair O101/O102, a pair of peaks with densities 0.48 and 0.42 e Å^−3^ appeared near the split O atom in positions compatible with the geometry of two partial O atoms sharing a single pair of H atoms. The maxima were assigned to and successfully refined as H atoms. A tentative split-atom description of O9 was not successful. However, this model revealed two residual maxima with densities 0.35 and 0.26 e Å^−3^ corresponding to one pair of H atoms bonded to the split O91/O92 atom, similar to the result obtained for the pair O101/O102. In the final stages of refinement only O10 was treated as a split atom pair, all other non-H atoms, except K1, were treated as single-site atoms and refined with ADPs. No restraints were applied.

The model with a split O10 atom led to small improvements in the bonding geometry involving some of the H atoms. At convergence, four of the five H—O—H bond angles were in the range 98–106°, the angle at O7 was 112°; of the 12 O—H bonds, nine were in the range 0.81–0.88 Å, the remainder were 0.75, 0.78 and 0.98 Å. Eight pairs of parameters associated either with K1 or with O10 had correlation coefficients greater than 0.8. However, the refinement was stable and final interatomic distances K1—K1^ii^ (at −*x*, −*y*, *z*) were 0.362 (4) Å and O101—O102 = 0.448 (6) Å. The three largest residual maxima, in the range 0.19–0.26 e Å^−3^, correspond to bonding density in the three C—C bonds of the tartrate molecule. The remaining residual extrema were in the range −0.19 to 0.19 e Å^−3^. Crystal data are given in Table 1 ▶. A survey of the final refinements is given in Table 2 ▶. Table 3 ▶ contains the final atomic parameters. A selection of bonding parameters, excluding those for the K and Na coordination shells, is shown in Table 4 ▶. Anisotropic displacement parameters and selected torsion angles are given in the supporting information.

Programs used for the various crystallographic operations were for diffractometer control and data collection: *KM4B8-KM4* diffractometer control and data collection program (Galdecki *et al.*, 1997 ▶); data reduction, scaling and absorption correction: *xd_red*-1.0 (Mathiesen, 2001 ▶); structure refinement: *SHELXL*97 (Sheldrick, 2008 ▶) and graphics: *ORTEP*3 (Farrugia, 1997 ▶).

## 3. Results   

### 3.1. ADPs and the disorder model   

The refinement showed unequivocally that atom K1 is disordered, residing in a general position with weight 0.5. Shiozaki *et al.* (2001 ▶) found evidence for a disordered K1 in both high-*T* and low-*T* PE structures within two wide ranges of *T*, *e.g.* as low as 153 K. They reported disorder in all the constituent atoms, but did not elaborate further on the disorder model. In a precise study of the low-*T* PE structure at 105 K, Görbitz & Sagstuen (2008 ▶) found no evidence of disorder for any of the atoms. In our study at 308 K, three of the four water O atoms O10, O9 and O8, display very strong anisotropy. Refining O10 as a split atom was feasible and yielded small improvements in bonding parameters involving several H atoms. Both O9 and O8 could be satisfactorily described as single-site atoms with ADPs. A crucial observation is that for each of these water molecules, the H atoms behave as a single pair that could be refined without restraints. The final *U* values, with averages of 0.056, 0.102 and 0.059 Å^2^ for the pairs H*n*10, H*n*9 and H*n*8, *n* = 1, 2, respectively, indicate that they do not or do only to a very small extent participate in the anisotropy of the O atoms to which they are bonded. The emerging picture is one in which the three O atoms are statically disordered, while the bonded H atoms are not. The difference in modelling the three O atoms is not significant, but rather reflects what is technically feasible within the harmonic approximation of the atom. Except for the split K1 and the three water O atoms there is nothing unusual about the ADPs of the remaining atoms, thus there is no indication of general disorder.

### 3.2. The structure   

**Figure 1:**
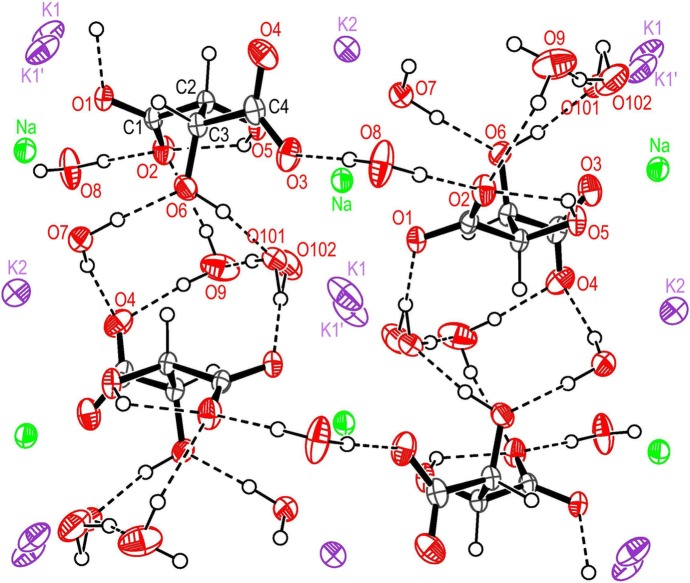
View of the structure approximately along [001] showing atomic numbering and the hydrogen bonding. The origin is at the upper left corner near the pair K1/K1′, the *y* axis is pointing to the right, *x* is pointing down. Displacement ellipsoids represent 50% probability. K1′ corresponds to K1^ii^ in Table 5 ▶.

A projection of the unit cell approximately along the *c* axis is shown in Fig. 1 ▶. A list of equivalent positions is given in Table 5 ▶ which correspond to the superscripts used in the text and in Tables 6 ▶ and 7 ▶. The tartrate molecules in Fig. 1 ▶ are in the l(+) configuration [later renamed (2*R*, 3*R*)] as was first established in a famous diffraction experiment by Bijvoet *et al.* (1951 ▶). The water molecules *W*7, *W*9 and *W*101/102 are engaged in closed hydrogen-bond loops connecting neighbour tartrate molecules in the *a* direction, there are also strong components along *c*. The *W*8 molecules are located together with the K and Na atoms in the channels running along **a** at *y* = *m*/2, *m* = 0, ±1, ±2, … . Atom O8 links adjacent chains of tartrate molecules across the channel by two hydrogen bonds. The main features of the crystal structure, even in details, are very similar to those found for the LT form at 105 K (Görbitz & Sagstuen, 2008 ▶).

#### 3.2.1. The hydrogen bonding   

Nearly all previous structure studies of RS from Beevers & Hughes (1941 ▶) on have tentatively linked the phase transition to disorder and/or dynamical changes in the hydrogen-bond system. It is therefore of great importance to study in detail the hydrogen-bonding geometry which is set out in Table 6 ▶. For all calculations of contact distances hydrogen bond lengths have been normalized to C—H = 1.10 Å and O—H = 0.985 Å. These distances are mean values corrected for thermal effects for C(*sp* ^3^)—H and O—H bonds, respectively, obtained from precise neutron diffraction studies (Koetzle *et al.*, 1972 ▶; Brown & Levy, 1965 ▶, 1973 ▶; Floriano *et al.*, 1987 ▶). O—H bonds in the LT form at 105 K (Görbitz & Sagstuen, 2008 ▶) have also been normalized and the geometry parameters are included in this table for comparison. In both structures, H5 is involved in a bifurcated hydrogen bond, the contact to O3^vii^ is rather long and weak. Görbitz & Sagstuen (2008 ▶) pointed out that H19 participates in a trifurcated hydrogen bond. This is also found in the structure at 308 K; the longest contact, to O3^xi^, is 2.681 Å *cf.* 2.504 Å in the 105 K structure. The long *D*⋯*A* contacts have been included in the table to emphasize the similarity of the two hydrogen-bond systems. The table indicates a small relative torsion of the fork of H19⋯O contacts in the two structures. For the remaining hydrogen bonds there is a close correspondence in geometry, H⋯*A* contacts at 105 K being consistently slightly shorter, on average −1.6%. The overall similarity is a remarkable result in view of the difference in *T* of about 200 K, involving a transition through the FE state.

#### 3.2.2. The coordination about K and Na   

**Figure 2:**
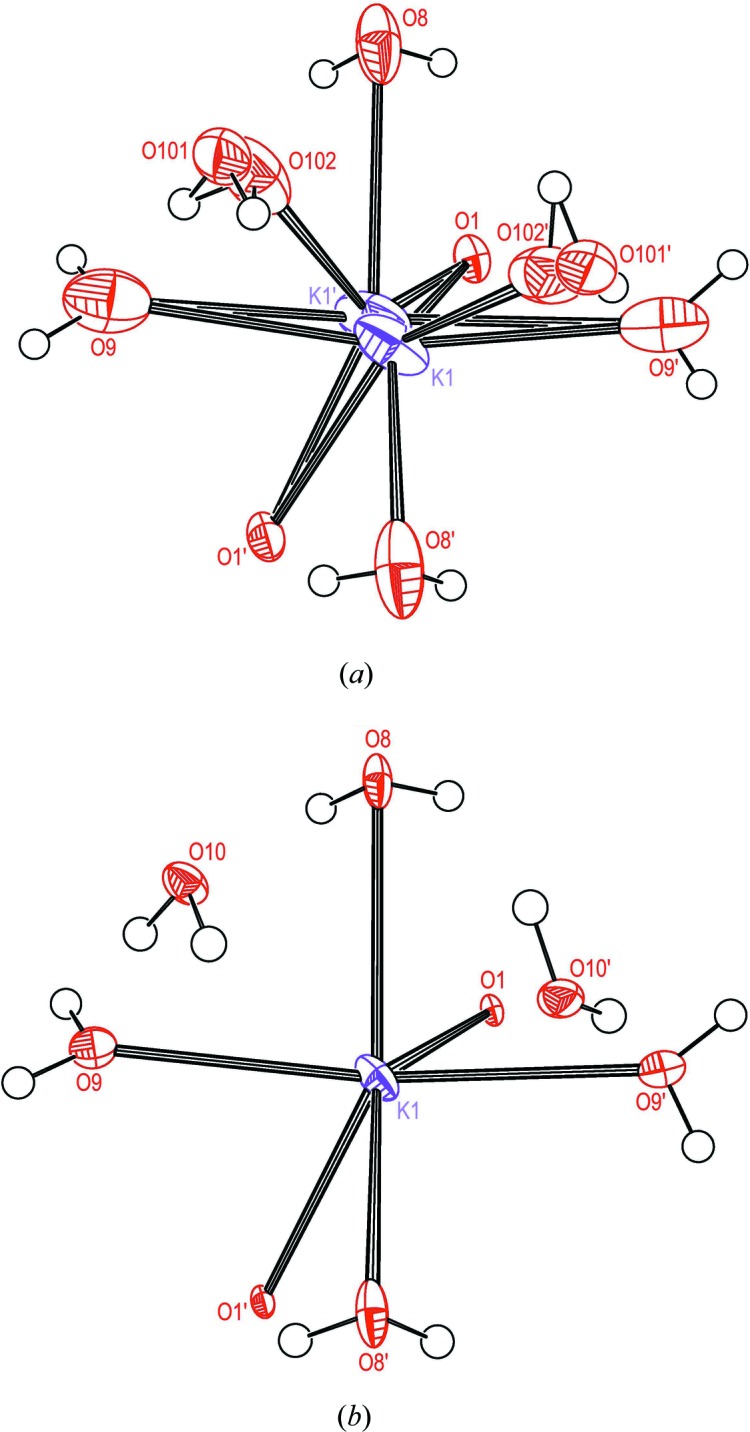
Ellipsoid plot of the O coordination shell about K1 for (*a*) the HT and (*b*) the LT forms; atomic parameters for the latter are from Görbitz & Sagstuen (2008 ▶). H atoms bonded to O in the shell are included. O10 in the LT form (*b*) is too far removed from the K atom (3.427 Å) to participate in the coordination.

The coordination shells about K1 are distinct in the two PE structures and therefore of particular interest. In the HT structure (Fig. 2 ▶ *a*) eight O atoms participate, among which are those of the three water molecules *W*8, *W*9 and *W*101/102. All contacts less than 3.11 Å are shown as bonds. Within a distance range 2.79–3.11 Å, symmetry-equivalent pairs of atoms O1 and O9 coordinate with both members of the disordered pair K1/K1^ii^. Each member of the pairs of O8 and of O102 makes contact with either K1 or K1^ii^. We observe that all of the water O atoms have the largest extension of their displacement ellipsoids directed approximately towards the split pair K1/K1^ii^ as if to enhance the K—O interactions. Strong anisotropy in these water molecules has been noted and discussed in several previous studies of RS (Suzuki & Shiozaki, 1996 ▶; Solans *et al.*, 1997 ▶; Shiozaki *et al.*, 1998 ▶, 1999 ▶). Apparently the directional relationship to the K1 site pointed out above has not been appreciated.

Fig. 2 ▶(*b*) shows the same coordination shell for the low-*T* PE structure plotted from the data of Görbitz & Sagstuen (2008 ▶). At 105 K there are only six O atoms within the same distance range from K1 as found at 308 K. K1 occupies the special position 0, 0, *z* and shows no disorder. The pair of atoms O10 has been included in the plot but these atoms are located 3.427 Å from K1 and do not participate in the coordination shell. Anisotropy of the atoms O8 and O9 is modest, but also at 105 K the largest extension of their displacement ellipsoid points approximately towards K1. A selection of geometric parameters for the coordination shells at both temperatures is given in Table 7 ▶.

**Figure 3:**
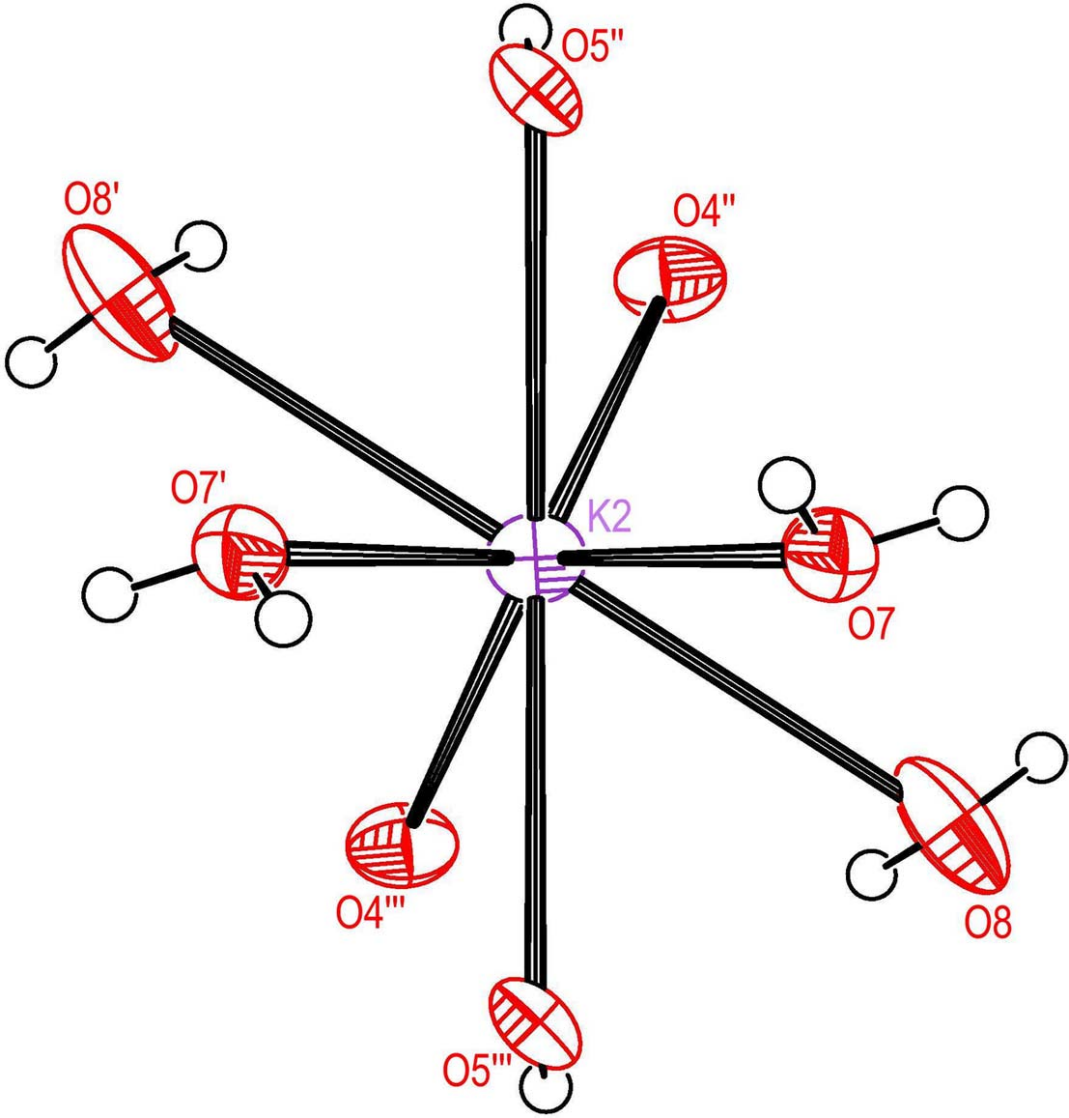
Ellipsoid plot of the O coordination shell about K2 for the HT form.

Figs. 3 ▶ and 4 ▶ are plots of the coordination shells about K2 and Na, respectively, for the high-*T* structure, parent coordination parameters are listed in Table 7 ▶. Eight O atoms within the distance range 2.81–3.12 Å contribute to the coordination about K2. The coordination about the Na atom comprises six O atoms in the distance range 2.33–2.51 Å. The coordination shells about K2 and Na at 105 K are closely similar to the high-*T* structure and therefore plots are not shown. At 308 K the distances from K2 to pairs of symmetry-equivalent atoms O*n*, *n* = 4, 5, 7, are on average 0.0245 Å longer than at 105 K; however, the K2—O8 distance is 0.052 Å shorter at 308 K. The average and maximum differences between the high-*T* and low-*T* structures in the four angles listed in Table 7 ▶ are 0.98 and 1.64°, respectively. The distances from Na to the six O atoms of the coordination shell are on average 0.0243 Å longer at 308 K than at 105 K. The average and maximum differences between the high-*T* and low-*T* PE structures in the three angles listed in Table 7 ▶ are 0.88 and 1.32°, respectively.

The unit-cell volume at 308 K has increased by 2.9% relative to that at 105 K. It appears that the space allotted to K1 at 105 K is sufficient to allow this atom to remain ordered and entertain a six-coordinated shell of O atoms. With increasing temperature the unit cell expands, at some *T* the K1 site is split in two, and the anisotropy of three of the water O atoms increases, presumably implying a certain degree of disorder, and the displacement ellipsoids of these atoms are significantly extended towards the disordered K1/K1^ii^ site. The combined effect is to secure an eight-atom coordination realised by a shift and presumably also a splitting of the O10 site to decrease the distance to K1/K1^ii^.

### 3.3. The impact of X-radiation on the PE structure   

It has long been known that RS is easily damaged by ionizing radiation. More specifically, the FE structure is gradually destroyed with increasing dose, as can be followed crystallographically by a change from monoclinic FE to the orthorhombic PE symmetry (Boutin *et al.*, 1963 ▶), and a concomitant decrease and disappearance of the hysteresis loops (of *P versus E*) and a blurring of the dielectric peak, features being associated with the loss of ferroelectricity (Okada *et al.*, 1967 ▶). As a salt of an organic dicarboxylic acid containing four waters of crystallization RS provides many sites for the creation of free radicals by ionizing radiation, and the crystals are susceptible to radiation also outside the FE range. Several studies applying various spectroscopic techniques have been carried out to explore the radical chemistry of X-ray irradiated or γ-irradiated single crystals of RS. A recent study by Sagstuen *et al.* (2012 ▶) using both spectroscopy and DFT calculations to characterize the primary-induced radicals at 10 K and secondary species formed at higher *T* also gives an excellent overview of previous work.

In our diffraction work at 308 K, we observed a significant increase ∼ 10σ in the unit-cell axis *b* and a smaller increase of 4σ in *c* during the period of irradiation that lasted about 70 h. Expansion of one or more unit-cell axes during X-ray irradiation is very often the mark of radiation damage. It has been suggested that the irreversible increase in *d* spacings (Müller *et al.*, 2002 ▶) or in unit-cell volume (Ravelli *et al.*, 2002 ▶) of organic and protein crystals could be used as a metric for radiation damage. In a time-resolved investigation of X-ray-induced damage in the sulfur amino acid taurine, both powder and single-crystal XRD were employed together with Raman spectroscopy (Beukes *et al.*, 2007 ▶). The most important results were a dose-dependent irreversible increase in the ADPs as well as in one of the unit-cell axes; furthermore, an enhancement of electron density in the SO_3_ group of the molecule that was tentatively attributed to primary radical formation involving this group. The observed changes were ascribed to the accumulation of foreign molecular species created by secondary reactions, thereby causing an expansion of the unit cell and local departure from crystalline order, *i.e.* enhanced static disorder and a build-up of local strain.

We believe the same mechanism is at play during X-ray exposure of RS. The creation of other molecular species following the primary formation of free radicals implicates repulsion and strain, as well as a need for expansion. The strongest intermolecular contacts are roughly confined to the *ac* plane. The cohesion along *b*, provided mainly by two hydrogen bonds with O8 as the donor and the coordination polyhedra about the K and Na atoms, is looser, making this direction the preferred one for expansion. The accumulation of debris, presumably in part being charged, may well be trapped preferentially in the channels running parallel to *a* at *y* = *m*/2, *m* = 0, ±1, ±2, … and will interact with the constituents of the structure, electrostatically and physically, thereby impeding or blocking completely the atomic translations involved in the phase transition. We found that with increasing radiation exposure the PE crystal does not transform into the FE phase, as seen below *T*_C2_ by the disappearance of 0*k*0 reflections for *k* odd, and split reflections again becoming or remaining single, both marks of the monodomain monoclinic PE crystal.

## 4. Conclusion   

For the structure study of the PE form of RS at 308 K, diffraction data of excellent quality were obtained by the use of a new gas-flow sample cell allowing control of relative humidity and temperature, which are critical parameters for the preservation of these crystals, in particular, under the impact of ionizing radiation. All atomic parameters, including those for isotropic H atoms, could be refined without restraints.

The work showed unequivocally that atom K1 is disordered, occupying two general symmetry-equivalent positions with weight 0.5. Three of the four water O atoms, O8, O9 and O10, display very strong anisotropy. O10 was successfully refined as a split atom. Both O8 and O9 could be satisfactorily described as single-site atoms with displacement ellipsoids. The three associated pairs of H atoms do not participate in the strong anisotropy of the O atoms to which they are bonded, suggesting that the disorder also of O8 and O9 may be in part of static nature. Except for the split K1 atom and the three water O atoms, there is no indication of general disorder in the structure.

Most structural features, including the hydrogen-bond system, are remarkably similar to those found in a precise diffraction study of the PE form at 105 K (Görbitz & Sagstuen, 2008 ▶). In the latter structure, however, there was no evidence of disorder. A striking difference pertains to the coordination about K1. At 308 K, symmetry-equivalent pairs of the atoms O1, O8, O9 and O101/O102 share in an eight-coordination polyhedron about the split pair K1/K1^ii^ with K—O distances in the range 2.79–3.11 Å. All of the water O atoms have the largest extension of their anisotropic displacement ellipsoids directed approximately towards the K1/K1^ii^ site, so as to enhance the K—O interactions. At 105 K, the pair of O10 atoms is displaced away from K1 to a distance of 2.427 Å, leaving only six O atoms in the coordination shell.

With increasing accumulation of radiation damage the PE crystal eventually does not transform into the FE phase.

## Supplementary Material

Crystal structure: contains datablock(s) RS_PE_308K. DOI: 10.1107/S2052252514022155/yu5002sup1.cif

Structure factors: contains datablock(s) f2shx. DOI: 10.1107/S2052252514022155/yu5002sup2.fcf

Two tables. DOI: 10.1107/S2052252514022155/yu5002sup3.doc

CCDC reference: 1030936

## Figures and Tables

**Figure 4:**
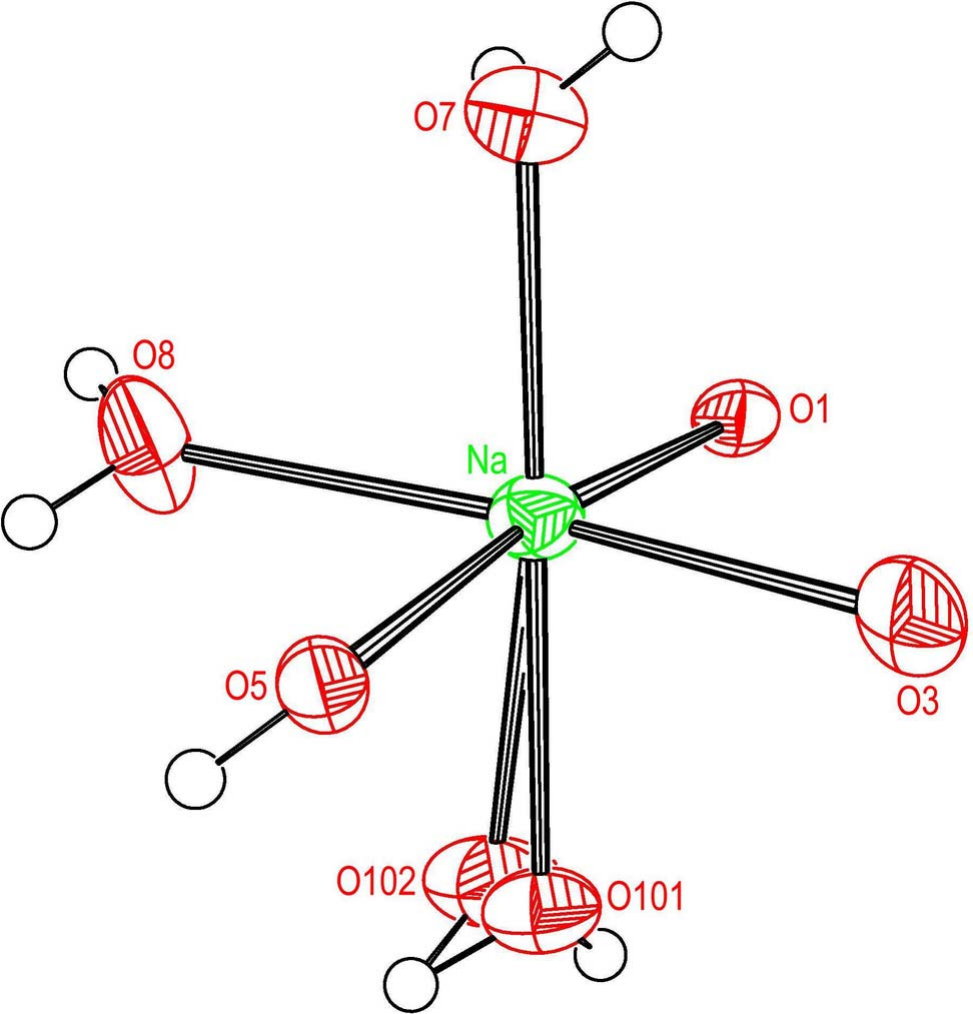
Ellipsoid plot of the O coordination shell about Na for the HT form.

**Table 1:** Crystallographic data

Composition	K^+^Na^+^ C_4_H_4_O_6_^2^4H_2_O
Formula weight, *M_r_*	282.22
Melting range/anhydrate/decomposition (K)	343353/403413/initial 493
Crystal system, space group	Orthorhombic, *P*2_1_2_1_2
Temperature, *T* (K)	308.0(7)
Unit-cell dimensions ()	*a* = 11.9247(5), *b* = 14.3066(7), *c* = 6.2444(5)
*V* (^3^)	1065.31(11)
Molecules per unit cell, *Z*	4
Calc. density, *D_x_* (Mgm^3^)	1.7596(2)
Wavelength, ()	0.60097(10)
Crystal size (mm)	0.20 0.14 0.088
Absorption coefficient, 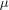 (mm^1^)	0.352
Transmission, min/max	0.952/0.977
TDS correction 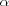 , min/max	1 10^5^/5.869 10^2^

**Table 2:** Data collection and processing, survey of refinements

Beam size (mm)	0.6 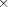 0.6
Scan mode	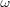
Scan range ()/ No. of scan steps	0.18/120
Resolution, *s*_high_/*s*_max_ 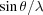 (^1^)	0.781/1.00
Completeness within *s*_high_/*s*_max_ (%)	100/54.5
Total No. of reflections/unique reflections	8753/4729
*R*_merge_(all)/*R*_merge_(obs)/*R*_sigma_(all)	0.0123/0.0121/0.0146
Unique reflections with *F* ^2^ > 2(*F* ^2^) (*NO*)	3879
No. reflections/restraints/variables (*NV*)	4711/0/207
Final (shift/e.s.d.), max/mean	0.019/0.002
Final *R* indices [*F* ^2^ > 2(*F* ^2^)]†	*R*(*F*) = 0.0236, *wR*(*F* ^2^) = 0.0579
Final *R* indices (all data)	*R*(*F*) = 0.0371, *wR*(*F* ^2^) = 0.0608
Weight parameters *w*_A_ and *w*_B_	0.0364/0.0
Goodness of fit (GOF) on *F* ^2^ ‡	0.997
Flack *x* parameter	0.008(31)
Non-bonding extrema in final electron density (e^3^)	0.19 to 0.19

† *w* = 1/[^2^(*F*_o_^2^) + (*w*_A_ *P*)^2^ + *w*_B_ *P*], where *P* = [max (*F*_o_^2^, 0) + 2*F*_c_^2^]/3; *R*(*F*) = ||*F*_o_| |*F*_c_||/|*F*_o_|; *wR*(*F* ^2^) = {[*w*(*F*_o_^2^ *F*_c_^2^)^2^]/[*w*(*F*_o_^2^)^2^]}^1/2^.

‡ GOF = [*w*(*F*_o_^2^ *F*_c_^2^)^2^/(*NO* *NV*)]^1/2^.

**Table 3:** Final atomic parameters Fractional coordinates, anisotropic displacement parameters *U*_eq_10^5^ (^2^) for non-H atoms and isotropic displacement parameters *U*10^4^ (^2^) for H atoms. E.s.d.’s in parentheses.

Atom	*x*	*y*	*z*	*U*_eq_ or *U* for H
K1	0.01429 (16)	0.00429 (22)	0.04853 (9)	5191 (41)
K2	0.5000	0.0000	0.15894 (5)	3477 (6)
Na	0.23125 (3)	0.00693 (3)	0.52355 (6)	2892 (7)
O1	0.11972 (6)	0.10915 (4)	0.35143 (11)	2753 (12)
O2	0.20925 (7)	0.20272 (4)	0.11931 (11)	3350 (14)
O3	0.23403 (8)	0.40621 (5)	0.81191 (12)	4002 (17)
O4	0.05413 (7)	0.36293 (5)	0.84314 (13)	3953 (16)
O5	0.16310 (6)	0.35702 (4)	0.32313 (10)	2847 (12)
O6	0.29526 (6)	0.24827 (5)	0.62716 (12)	3156 (14)
O7	0.39539 (6)	0.08237 (5)	0.48436 (14)	3614 (16)
O8	0.24463 (10)	0.04106 (6)	0.88637 (13)	5175 (26)
O9	0.06208 (9)	0.20000 (10)	0.03530 (20)	6164 (29)
O101	0.07845 (39)	0.11072 (25)	0.56295 (75)	3666 (57)
O102	0.06835 (46)	0.08943 (27)	0.61190 (84)	5756 (115)
C1	0.15406 (7)	0.18766 (5)	0.28517 (13)	2178 (13)
C2	0.12476 (7)	0.27338 (5)	0.42311 (12)	2140 (12)
C3	0.17820 (7)	0.26422 (5)	0.64501 (13)	2284 (13)
C4	0.15300 (9)	0.35190 (6)	0.77916 (13)	2776 (16)
H2	0.0423 (11)	0.2761 (9)	0.4377 (22)	333 (31)
H3	0.1454 (10)	0.2101 (8)	0.7235 (21)	242 (27)
H5	0.1926 (15)	0.3376 (12)	0.2047 (35)	608 (50)
H6	0.3281 (15)	0.2945 (13)	0.6096 (33)	591 (51)
H17	0.3621 (14)	0.1324 (12)	0.5170 (27)	486 (41)
H27	0.4348 (15)	0.0886 (11)	0.3800 (30)	581 (50)
H18	0.2530 (13)	0.0016 (15)	0.9895 (28)	602 (46)
H28	0.2319 (15)	0.0912 (14)	0.9524 (27)	573 (45)
H19	0.1174 (26)	0.2193 (20)	0.0016 (44)	1019 (89)
H29	0.0261 (23)	0.2592 (16)	0.0739 (41)	1020 (81)
H110	0.0135 (17)	0.1009 (13)	0.5231 (27)	585 (47)
H210	0.0737 (15)	0.1386 (12)	0.6880 (30)	537 (46)

**Table 4:** Bond lengths () and bond angles () in the tartrate molecule and the water molecules Bond parameters for the K and Na coordination shells are given in Table 7 ▶.

O1C1	1.265 (1)	O6H6	0.78 (2)
O2C1	1.246 (1)	O7H17	0.84 (2)
O3C4	1.257 (1)	O7H27	0.81 (2)
O4C4	1.255 (1)	O8H18	0.86 (2)
O5C2	1.425 (1)	O8H28	0.84 (2)
O6C3	1.419 (1)	O9H19	0.75 (3)
C1C2	1.539 (1)	O9H29	0.98 (2)
C2C3	1.531 (1)	O101H110	0.83 (2)
C3C4	1.538 (1)	O101H210	0.88 (2)
C2H2	0.99 (1)	O102H110	0.87 (2)
C3H3	1.00 (1)	O102H210	0.85 (2)
O5H5	0.86 (2)		

O2C1O1	126.62 (7)	O5C2H2	109.1 (8)
O2C1C2	116.57 (7)	C3C2H2	109.5 (8)
O1C1C2	116.81 (7)	C1C2H2	108.0 (8)
O5C2C3	109.57 (6)	O6C3H3	107.4 (7)
O5C2 C1	110.56 (6)	C2C3H3	110.4 (7)
C1C2 C3	110.11 (6)	C4C3H3	106.8 (7)
O6C3C2	110.62 (7)	H17O7H27	112.0 (1.5)
O6C3C4	111.48 (7)	H18O8H28	102.4 (1.7)
C2C3C4	109.99 (6)	H19O9H29	98.2 (2.5)
O3C4O4	126.36 (8)	H110O101H210	106.5 (1.6)
O3C4C3	116.28 (9)	H110O102H210	104.8 (1.7)
O4C4C3	117.36 (8)		

**Table 5:** Symmetry operations generating equivalent positions

(i)	*x*, *y*, *z* + 1	(viii)	*x*, *y*, *z* 1
(ii)	*x*, *y*, *z*	(ix)	*x* + 1, *y*, *z* 1
(iii)	*x*, *y*, *z* + 1	(x)	*x* + *, y +* , *z* + 1
(iv)	*x* + , *y* + , *z* + 1	(xi)	*x* + , *y* , *z* + 1
(v)	*x* , *y* + , *z*	(xii)	*x* + , *y* + , *z*
(vi)	*x* , *y* + , *z* + 1	(xiii)	*x* + , *y* , *z*
(vii)	*x*, *y*, *z* 1	(xiv)	*x* + , *y* , *z* + 2

**Table 6:** Hydrogen-bond geometry Hydrogen-bonding geometry in the LT form at 105K (Grbitz Sagstuen, 2008 ▶), in italics, is included in the table for comparison. All contacts have been recalculated with OH distances normalized to 0.985. The symmetry operations are given in Table 5 ▶

	H*A* ()		*D* *A* ()		*D*H*A* ()	
*D*H*A*	308K	105K	308K	105K†	308K	105K
O5H5O2	1.946	*1.917*	2.607 (1)	*2.595*	122.1	*123.5*
O5H5O3^vii^	2.600	*2.528*	3.377 (1)	*3.282*	135.8	*133.1*
O6H6O101^x^	1.872	*1.847* ‡	2.783 (3)	*2.812* ‡	152.6	*165.8* ‡
O6H6O102^x^	2.304		3.204 (4)		151.5	
O7H17O6	1.823	*1.799*	2.803 (1)	*2.783*	172.2	*176.5*
O7H27O4^iv^	1.940	*1.868*	2.894 (1)	*2.850*	162.6	*174.4*
O8H18O3^xiv^	1.728	*1.714*	2.709 (1)	*2.694*	172.8	*173.0*
O8H28O2^i^	1.781	*1.768*	2.765 (1)	*2.750*	175.7	*175.2*
O9H19O2^xiii^	2.282	*2.410*	3.106 (2)	*3.118*	140.5	*128.4*
O9H19O6^xi^	2.428	*2.279*	3.152 (1)	*3.103*	130.0	*140.5*
O9H19O3^xi^	2.681	*2.504*	3.189 (2)	*3.157*	112.4	*123.6*
O9H29O4^viii^	1.835	*1.803*	2.816 (2)	*2.784*	173.9	*173.4*
O101H110O1^ii^	1.786	*1.770* §	2.707 (5)	*2.71*2§	157.5	*158.9 * §
O102H210O9^i^	1.833	*1.796 * ¶	2.713 (6)	*2.753* ¶	147.8	*163.1* ¶

† E.s.d.’s in the range (0.60.8) 10^3^.

‡ O6H6O10^x^.

§ O10H110O1^ii^.

¶ O10H210O9^i^.

**Table 7:** Coordination shell geometries (, ) Parameters for the coordination shell about K1 in the LT form at 105K (Grbitz Sagstuen, 2008 ▶) in italics. Angles with K1^ii^ at the apex are identical by symmetry.

Coordination shell about K1				Coordination shell about K2		Coordination shell about Na	
308K		105K		308K		308K	
K1O1	2.895 (2)		*2.8194*	K2O7^vii^	2.8118 (9)	NaO102	2.3391 (55)
K1O1^ii^	2.791 (2)		*2.8194*	K2 O7^ix^	2.8118 (9)	NaO101	2.3633 (46)
K1^ii^O1	2.791 (2)			K2O4^iv^	2.8550 (8)	NaO7	2.3502 (8)
K1O1^ii^	2.895 (2)			K2O4^xi^	2.8550 (8)	NaO8	2.3727 (9)
K1O9	3.106 (3)		*2.8491*	K2O5^xii^	3.0030 (7)	NaO1	2.3837 (7)
K1O9^ii^	2.905 (3)		*2.8491*	K2O5^xiii^	3.0030 (7)	NaO3^xi^	2.4705 (9)
K1^ii^O9	2.905 (3)			K2O8^vii^	3.1143 (12)	NaO5^xi^	2.5085 (7)
K1O9^ii^	3.106 (3)			K2O8^ix^	3.1143 (12)		
K1O8^viii^	2.998 (2)		*3.0271*				
K1^ii^O8^vii^	2.998 (2)	K1O8^vii^	*3.0271*				
K1O102^viii^	3.055 (5)	K1O10^viii^	*3.4266*				
K1^ii^O102^vii^	3.055 (5)	K1O10^vii^	*3.4266*				
							
O102^vii^K1O102^viii^	58.04 (18)	O10K1O10^ii^	*62.98*	O7^vii^K2O7^ix^	75.22 (3)	O102NaO7	172.12 (12)
				O4^iv^K2O4^xi^	92.63 (4)	O101NaO7	173.99 (10)
O1K1O1^ii^	96.15 (3)		*95.81*	O5^xii^K2O5^xiii^	140.07 (3)	O1NaO5^xi^	173.17 (3)
O8^vii^K1O8^viii^	142.18 (4)		*141.00*	O8^vii^K2O8^ix^	169.58 (3)	O8NaO3^xi^	160.72 (3)
O9K1O9^ii^	159.10 (5)		*161.78*				
