# Supplementary material for: Rochelle salt – a structural reinvestigation with improved tools. I. The high-temperature para­electric phase at 308 K
Source: IUCrJ. 2015 Jan 1;2(Pt 1):19–28. doi: 10.1107/S2052252514022155 (PMC4285877; doi:10.1107/S2052252514022155)
Supplement: Supplementary file 3 [file m-02-00019-sup3.doc]

Supporting information for article:

# Rochelle salt – a structural reinvestigation with improved tools

# The high-*T* paraelectric phase at 308 K

# Frode Mo,* Ragnvald H. Mathiesen, Jon Are Beukes^a^ and Khanh Minh Vu

# Department of Physics, Norwegian University of Science and Technology, N-7491, Trondheim, Norway

^a^ Present address: Schenck Process Norge AS, Bedriftsveien 25, N-4313 Sandnes, Norway.

# Correspondence e-mail: frode.mo@ntnu.no

**Table S1** *Anisotropic displacement parameters (Å^2^) with esd’s.*

*U*_11_ *U*_22_ *U*_33_ *U*_12_ *U*_13_ *U*_23_

K1 0.0508(10) 0.0588(5) 0.0462(2) -0.0272(7) -0.0088(3) 0.0130(5)

K2 0.03621(13) 0.03682(13) 0.03128(12) -0.00428(11) 0.000 0.000

Na 0.03033(16) 0.02338(14) 0.03305(16) -0.00073(13) -0.00194(14) 0.00124(14)

O1 0.0341(3) 0.0176(2) 0.0309(3) -0.0013(2) -0.0011(3) -0.0005(2)

O2 0.0483(4) 0.0263(3) 0.0260(3) 0.0013(3) 0.0085(3) -0.0019(2)

O3 0.0554(5) 0.0283(3) 0.0363(4) -0.0075(3) -0.0035(3) -0.0087(3)

O4 0.0435(4) 0.0429(4) 0.0323(3) 0.0124(3) 0.0015(3) -0.0088(3)

O5 0.0452(4) 0.0170(2) 0.0232(3) -0.0005(2) 0.0000(3) 0.0012(2)

O6 0.0301(3) 0.0261(3) 0.0385(4) 0.0056(2) -0.0049(3) -0.0022(3)

O7 0.0329(3) 0.0299(3) 0.0456(4) -0.0007(3) 0.0065(3) 0.0018(3)

O8 0.1030(8) 0.0262(3) 0.0261(3) 0.0129(4) 0.0066(4) 0.0001(3)

O9 0.0410(5) 0.0888(9) 0.0552(6) -0.0101(5) -0.0108(5) 0.0078(6)

O101 0.0269(8) 0.0315(12) 0.0516(14) -0.0046(9) -0.0019(9) 0.0008(9)

O102 0.0460(19) 0.047(2) 0.080(3) -0.0166(15) -0.024(2) 0.0229(17)

C1 0.0262(3) 0.0179(2) 0.0212(3) 0.0017(2) -0.0027(3) -0.0021(2)

C2 0.0270(3) 0.0168(2) 0.0204(3) 0.0014(2) -0.0009(3) -0.0011(2)

C3 0.0297(3) 0.0185(3) 0.0203(3) 0.0019(2) -0.0014(3) -0.0005(2)

C4 0.0415(5) 0.0227(3) 0.0190(3) 0.0046(3) -0.0037(3) -0.0026(3)

**Table S2** *Torsion angles (^o^) with esd’s* *of the tartrate moiety.*

O2 – C1 – C2 – O5 3.27 (10) O5 – C2 – C3 – C4 55.77 (9)

O1 – C1 – C2 – O5 -176.70 (7) C1 – C2 – C3 – C4 177.58 (7)

O2 – C1 – C2 – C3 -117.95 (8) O6 – C3 – C4 – O3 14.89 (11)

O1 – C1 – C2 – C3 62.08 (9) C2 – C3 – C4 – O3 -108.19 (8)

O5 – C2 – C3 – O6 -67.81 (8) O6 – C3 – C4 – O4 -165.84 (8)

C1 – C2 – C3 – O6 54.00 (8) C2 – C3 – C4 – O4 71.07 (10)
